# A transparent 13-gene transcriptomic candidate signature for cervical neoplastic spectrum across GEO cohorts

**DOI:** 10.3389/fgene.2026.1875050

**Published:** 2026-10-30

**Authors:** Pengbo Zhao, Huixia Lu

**Affiliations:** Clinical Medical College, Dali University, Dali, Yunnan, China

**Keywords:** calibration, cervical cancer, cervical neoplasia, GEO, machine learning, nested cross-validation, SMPD2, transcriptomics

## Abstract

Transcriptomic classifiers can prioritize molecular signals associated with cervical neoplastic lesions, but high-dimensional feature selection is vulnerable to overfitting, information leakage, and cohort-selection bias. We performed a retrospective computational reanalysis of six Gene Expression Omnibus microarray cohorts retained from the original project, with cervical tissue or epithelial expression profiles, extractable binary labels, and harmonizable gene annotation. GSE63514, GSE67522, and GSE9750 formed the development cohort (n = 200; 127 case samples and 73 control samples), whereas GSE64217, GSE6791, and GSE7803 were reserved for external evaluation. A glmBoost-derived 13-gene panel was coupled to a gradient boosting machine (GBM). The supervised stages of internal evaluation used 10 repeats of stratified five-fold nested cross-validation; scaling, feature selection, and GBM tuning were repeated within every outer-training split. The inherited expression matrix had undergone cohort-wide ComBat adjustment before this reconstruction, so the analysis does not establish fully end-to-end foldwise preprocessing validation. The aggregated out-of-fold AUC for the reconstructed selection pipeline was 0.9161 (95% CI 0.8731–0.9591). External discrimination was high but cohort dependent; the nominal AUC of 1.0000 in the six-sample GSE64217 cohort was too imprecise to support a claim of perfect performance. Five genes were selected in all 50 outer models. In a *post hoc* fixed-panel sensitivity analysis, the 13-gene and five-gene panels had similar repeated-CV out-of-fold AUCs (0.924 and 0.931; paired DeLong P = 0.390). The signature is therefore a candidate computational model for a heterogeneous cervical neoplastic spectrum. It does not establish biological validation, comparative clinical utility, or a deployable single-patient assay.

## Introduction

Cervical cancer prevention has been transformed by HPV vaccination, HPV testing, cytology, colposcopy, and treatment of precursor lesions, yet clinically actionable triage remains difficult in many settings (Arbyn et al., 2020; Bruni et al., 2016; Sung et al., 2021); Wentzensen et al., 2017. Persistent infection with carcinogenic HPV types is the necessary biological cause of nearly all cervical cancers, but infection is common and most infections do not progress to invasive disease (Walboomers et al., 1999; Bosch et al., 2002; Burd, 2003; Schiffman et al., 2007; de Sanjose et al., 2010; Doorbar et al., 2012; Crosbie et al., 2013; Schiffman et al., 2016). Molecular signatures derived from cervical tissue or exfoliated cells may eventually complement established screening and diagnostic pathways, but only if they demonstrate reproducible discrimination, calibration, analytical validity, clinical utility, and incremental value beyond established HPV-based strategies (Ronco et al., 2014; Koliopoulos et al., 2017; Arbyn et al., 2018; Fontham et al., 2020; World Health Organization, 2021).

Public transcriptomic repositories provide an attractive route for discovering disease-associated expression patterns across independent cervical studies (Edgar et al., 2002; Barrett et al., 2013). Their value, however, depends on whether apparent biomarker performance survives the methodological pressures that commonly affect multi-cohort modeling: batch effects, heterogeneous case definitions, small validation sets, class imbalance, and information leakage when feature selection or model choice uses validation information (Ambroise and McLachlan, 2002; Johnson et al., 2007; Leek et al., 2012; Varma and Simon, 2006). Prediction-model guidance requires every data-dependent step to be repeated within resampling and prohibits use of external outcomes for hyperparameter tuning or model selection (Harrell et al., 1996; Steyerberg et al., 2010; Moons et al., 2015; Steyerberg and Harrell, 2016; Wolff et al., 2019; Riley et al., 2020; Collins et al., 2024). Reporting AUC alone is also insufficient; uncertainty, threshold-dependent performance, calibration, intended clinical role, and decision consequences should be described before a model is considered clinically useful (Brier, 1950; Van Calster et al., 2019; Vickers and Elkin, 2006).

The initial analysis screened 121 named machine-learning configurations and produced a 13-gene model, but code review showed that the operational pipeline was narrower than the model label implied: glmBoost feature selection followed by GBM classification, with the third component in the original glmBoost + GBM + Lasso label not executed. The audit also identified two sources of optimism: external-cohort AUCs had entered the model-ranking display, and the reported training AUC was a resubstitution estimate. We therefore reconstructed supervised model evaluation with development/external separation, repeated nested cross-validation, and a locked threshold. This reconstruction cannot retrospectively supply a formal, date-stamped cohort-selection protocol or turn globally batch-adjusted public matrices into a fully deployment-ready preprocessing pipeline. Our objective was to quantify a bounded computational signal, its feature stability, and the evidence still required for biological, analytical, and clinical validation.

## Materials and methods

### Study design and GEO cohort selection

This strictly computational retrospective public-database study reanalyzed six GEO series retained in the original project. The eligibility framework for retaining a series was: human cervical tissue or cervical epithelial expression profiling by microarray; extractable case and control labels in the processed expression matrix; gene-level annotation that could be harmonized across cohorts; and sufficient overlap with the candidate-gene matrix. Samples without a binary label in the processed expression matrices, samples lacking the required gene-feature overlap, and series that could not be harmonized at gene-symbol level were not included. The original project did not retain a formal date-stamped GEO search log, so dataset selection is reported as a reproducibility and selection-bias limitation rather than reconstructed retrospectively.

GSE63514, GSE67522, and GSE9750 were merged as the development cohort because they provided the largest harmonizable set of labelled samples among the series retained in the original project. GSE64217, GSE6791, and GSE7803 were reserved for external evaluation because they were processed as independent GEO series outside the merged development matrix. The corresponding source-series publication or GEO accession record is provided in the final column of Table 1. For this reconstruction, external outcomes were not used to tune the model, select the probability threshold, or choose among alternative algorithms. No additional GEO cohort was added after examining model performance. The original project did not retain a formal date-stamped selection protocol; the cohort set should therefore be interpreted as a transparent reanalysis of inherited data rather than as an exhaustive or prospectively specified GEO survey. The analyzed sample counts and source-series characteristics are shown in Table 1.

**TABLE 1:** GEO cohorts and analyzed sample composition.

Cohort	Role	Platform	n	Disease	Control	Source-series context
GSE63514	Development	GPL570	92	68	24	Laser-captured cervical epithelium; processed case group includes CIN3 and cancer (source: den Boon et al., 2015)
GSE67522	Development	GPL10558	42	20	22	Cervical SCC and histologically normal cervical tissue; heterogeneous HPV status (source: Sharma et al., 2015)
GSE9750	Development	GPL96	66	39	27	Source series includes primary tumors, cervical cancer cell lines, and normal cervix (source: Scotto et al., 2008)
GSE64217	External	GPL10558	6	4	2	Normal cervix, CIN, and SCC represented; very small analyzed set (source: GEO accession record)
GSE6791	External	GPL570	28	20	8	Laser-capture-enriched cervical cancer and cervical normal epithelium (source: Pyeon et al., 2007 )
GSE7803	External	GPL96	41	32	9	Source series includes normal epithelium, HSIL, invasive SCC, and test samples (source: Zhai et al., 2007)

Case and control counts are the labels analyzed in the processed expression matrices. Several source series contain precursor lesions or other heterogeneous sample types; see Methods and Discussion.

The binary labels reflect the processed expression matrices. They are not clinically homogeneous across series: some case groups include cervical intraepithelial neoplasia (CIN) or high-grade squamous intraepithelial lesions (HSIL) together with invasive cervical cancer, and GSE9750 includes both primary tissues and cervical cancer cell-line material in the source series. The classifier therefore addresses a cervical neoplastic spectrum rather than a pure invasive-cancer-versus-healthy-tissue contrast. This endpoint creates possible spectrum bias, class ambiguity, label noise, and limits to transportability across screening, triage, diagnostic, and histology-specific clinical settings.

### Expression preprocessing and differential expression

Platform probe identifiers were mapped to gene symbols using the corresponding GEO platform annotation. When multiple probes mapped to one gene symbol, values were averaged. The three development expression matrices were intersected by gene symbol and combined. The inherited development matrix was batch-adjusted using the empirical-Bayes ComBat approach with GEO series as the batch variable (Bolstad et al., 2003; Irizarry et al., 2003; Johnson et al., 2007; Leek et al., 2012). Quantitative preprocessing quality control is now provided in Supplementary Figure S2C and Supplementary Table S6. These values quantify reduced GEO-series separation after ComBat, but do not prove preservation of biological signal or eliminate confounding.

The reconstructed source workflow estimated ComBat parameters on the combined development cohort before the present nested-validation reconstruction. We retained the inherited batch-adjusted matrix to make the audit reproducible, but this chronology means that the analysis does not claim fully foldwise ComBat preprocessing or completely leakage-free end-to-end processing. The nested stages that were rerun within outer-training folds were scaling, glmBoost selection, GBM fitting, and tree-number tuning. A future validation should estimate any batch harmonization strictly within training data or use a frozen deployment-compatible reference procedure.

Differential expression was assessed in the development cohort using limma (Ritchie et al., 2015). Genes with absolute log2 fold change greater than 0.585, corresponding to an approximately 1.5-fold expression difference, and Benjamini–Hochberg false-discovery-rate (FDR) adjusted P < 0.05 were considered differentially expressed (Benjamini and Hochberg, 1995). The original 1.5-fold threshold was selected to retain moderate transcriptomic changes for descriptive candidate-biology analysis rather than to define the classifier. As a sensitivity check, we recalculated descriptive counts using an absolute log2 fold-change threshold of 1.0 (two-fold) with the same FDR criterion; the fixed 13-gene classifier was not reselected or retrained at either threshold (Supplementary Table S7). Volcano plots and a heatmap of leading differentially expressed genes were generated without individual sample labels to improve readability.

### Functional enrichment

For exploratory biological interpretation, the candidate-gene set retained in the initial workflow was evaluated using Gene Ontology (GO) and Kyoto Encyclopedia of Genes and Genomes (KEGG) annotations (Ashburner et al., 2000; Gene Ontology Consortium, 2021; Kanehisa and Goto, 2000; Kanehisa et al., 2021). The clusterProfiler framework was used for enrichment analysis (Yu et al., 2012), and FDR-adjusted P < 0.05 defined significant enrichment. GO biological process, cellular component, and molecular function terms were reported separately. These enrichment analyses were used only to annotate biological context and were not used to select, tune, or validate the final 13-gene glmBoost-GBM classifier.

### Machine-learning pipeline audit and corrected model definition

The analysis code listed 121 model names assembled from penalized regression, stepwise regression, support vector machine, ridge regression, glmBoost, elastic net, random forest, GBM, XGBoost, naive Bayes, and linear discriminant analysis. Although these algorithm families and feature-selection strategies are established, their apparent performance can be optimistic when screening and validation are not separated (Cortes and Vapnik, 1995; Tibshirani, 1996; Breiman, 2001; Friedman, 2001; Guyon and Elisseeff, 2003; Buhlmann and Hothorn, 2007; Friedman et al., 2010; Chen and Guestrin, 2016). Inspection of the implementation showed that the first component of a multi-component name performed variable selection and the second fitted the classifier; subsequent components were ignored. The reported glmBoost + GBM + Lasso configuration was therefore operationally glmBoost feature selection followed by GBM classification. Its selected genes and predictions were nearly identical to those generated by the separately named glmBoost + GBM configuration. We consequently refer to the reconstructed supervised pipeline as glmBoost-GBM, while explicitly distinguishing it from the inherited cohort-wide ComBat preprocessing.

The 121-model matrix is retained in Supplementary Table S1 as an exploratory audit record. It is not presented as an unbiased comparison because external-cohort AUCs were included in the initial ranking. The reconstructed nested analysis focuses on the actual glmBoost-GBM pipeline and does not use external outcomes for the reconstructed model-selection stages.

### Repeated nested internal validation

Internal validation used 10 repeats of stratified five-fold outer cross-validation (50 outer models). Stratification was used to preserve the moderate case-control imbalance of the development cohort across folds. Within each outer split, means and standard deviations were estimated from the outer-training samples and applied to the held-out fold. GlmBoost feature selection was then repeated in the outer-training data, and GBM tuning was performed without access to the held-out samples. The inherited ComBat transformation had already been estimated on the combined development matrix before this reconstruction; therefore, the nested procedure protects scaling, feature selection, tuning, and evaluation from supervised leakage but does not demonstrate a fully foldwise batch-correction pipeline. No additional class weighting was applied during GBM fitting; threshold-dependent metrics were reported alongside AUC to show the effect of the observed class distribution.

Out-of-fold probabilities were averaged across the 10 repeats for each sample. The primary internal AUC and DeLong 95% confidence interval were calculated from these aggregated out-of-fold probabilities. The decision cutoff was selected once from the aggregated out-of-fold predictions using the Youden index. Sensitivity, specificity, accuracy, positive predictive value (PPV), negative predictive value (NPV), Brier score, calibration intercept, and calibration slope were calculated at this cutoff. In a separate *post hoc* fixed-panel sensitivity analysis, 10-bin Expected Calibration Error and loess-based Integrated Calibration Index were calculated for the five- and 13-gene panels; these choices were not prespecified in the original source workflow and are reported as exploratory.

### Locked final model and external evaluation

After internal validation, the development-derived 13-gene panel was frozen: SMPD2, VEGFA, AKR1B1, EED, TM7SF2, HEXA, FMO3, GSTA1, NEU1, ADM, TNNI2, CD207, and LBR. A GBM was refitted to all 200 standardized development samples. Five-fold cross-validation within the development cohort selected 4,192 trees; all other GBM parameters were retained from the reconstructed source workflow rather than selected by an expanded grid search, random search, or Bayesian optimization. The random seed was 123. The out-of-fold cutoff of 0.6136 was applied unchanged to external predictions.

The primary retrospective external analysis followed the initial platform-harmonization approach by z-standardizing genes within each external cohort before applying the locked classifier. A sensitivity analysis instead applied the development-set means and standard deviations unchanged to each external cohort. Cohort-wise standardization uses information from the full external cohort and is therefore transductive; it supports retrospective cohort comparison but is not a deployable single-patient procedure. Future single-sample implementation would require a locked normalization strategy, such as reference-gene normalization, frozen microarray normalization, or another platform-specific reference-based approach, before any clinical assay could be tested.

### ROC analysis, uncertainty, and model interpretation

AUCs and DeLong 95% confidence intervals were calculated with pROC (DeLong et al., 1988; Robin et al., 2011). Exact binomial 95% confidence intervals were calculated for sensitivity and specificity. A sensitivity analysis pooled GSE6791 and GSE7803 while excluding the six-sample GSE64217 cohort. Single-gene AUCs were direction-standardized for descriptive ranking only, so each value represented the better of the two expression directions; these single-gene summaries were not used to train or select the locked multigene classifier. An exploratory decision-analytic display was calculated from repeated-CV out-of-fold probabilities for the fixed five- and 13-gene panels (Supplementary Figure S2B). Because no target clinical decision, action threshold, comparator test, or prospective outcome was defined, it is not interpreted as evidence of clinical utility, a clinical impact curve, or net reclassification benefit.

Model interpretation used model-agnostic kernel SHAP values for a seed-fixed stratified subset of 60 development samples, with 20 development samples as the background distribution. SHAP values provide additive local feature attributions, while global summaries require careful interpretation in correlated biological data (Lundberg and Lee, 2017; Lundberg et al., 2020). Mean absolute SHAP values summarized global feature contribution. SHAP results were considered descriptive and did not establish causal biological effects.

### Software and reproducibility

Analyses were performed in R 4.5.0. Key packages included limma, sva/ComBat, mboost, gbm, caret (Kuhn, 2008), pROC, kernelshap, ggplot2, and patchwork. The accompanying files include the Reviewer three sensitivity-analysis script, derived out-of-fold predictions, summary tables, and figure-generation code. The principal random seed for the original reconstructed model was 123; the new sensitivity analysis used seed 20,260,727.

## Results

### Development-cohort composition and expression discovery

The analysis started from a 200-sample development cohort and three reserved external GEO matrices. The development cohort contained 127 case samples and 73 control samples, whereas the external matrices contained 4 case samples and two control samples in GSE64217, 20 and eight in GSE6791, and 32 and nine in GSE7803, respectively (Table 1). The six cohorts were obtained from GEO and their source studies or repository record are cited explicitly: GSE63514 (den Boon et al., 2015), GSE67522 (Sharma et al., 2015), GSE9750 (Scotto et al., 2008), GSE64217 (GEO accession record), GSE6791 (Pyeon et al., 2007), and GSE7803 (Zhai et al., 2007), together with the GEO repository citations (Edgar et al., 2002; Barrett et al., 2013). The workflow separates model development, nested supervised validation, model locking, and external evaluation (Figure 1), while the Discussion specifies the inherited batch-processing limitation.

**FIGURE 1:**
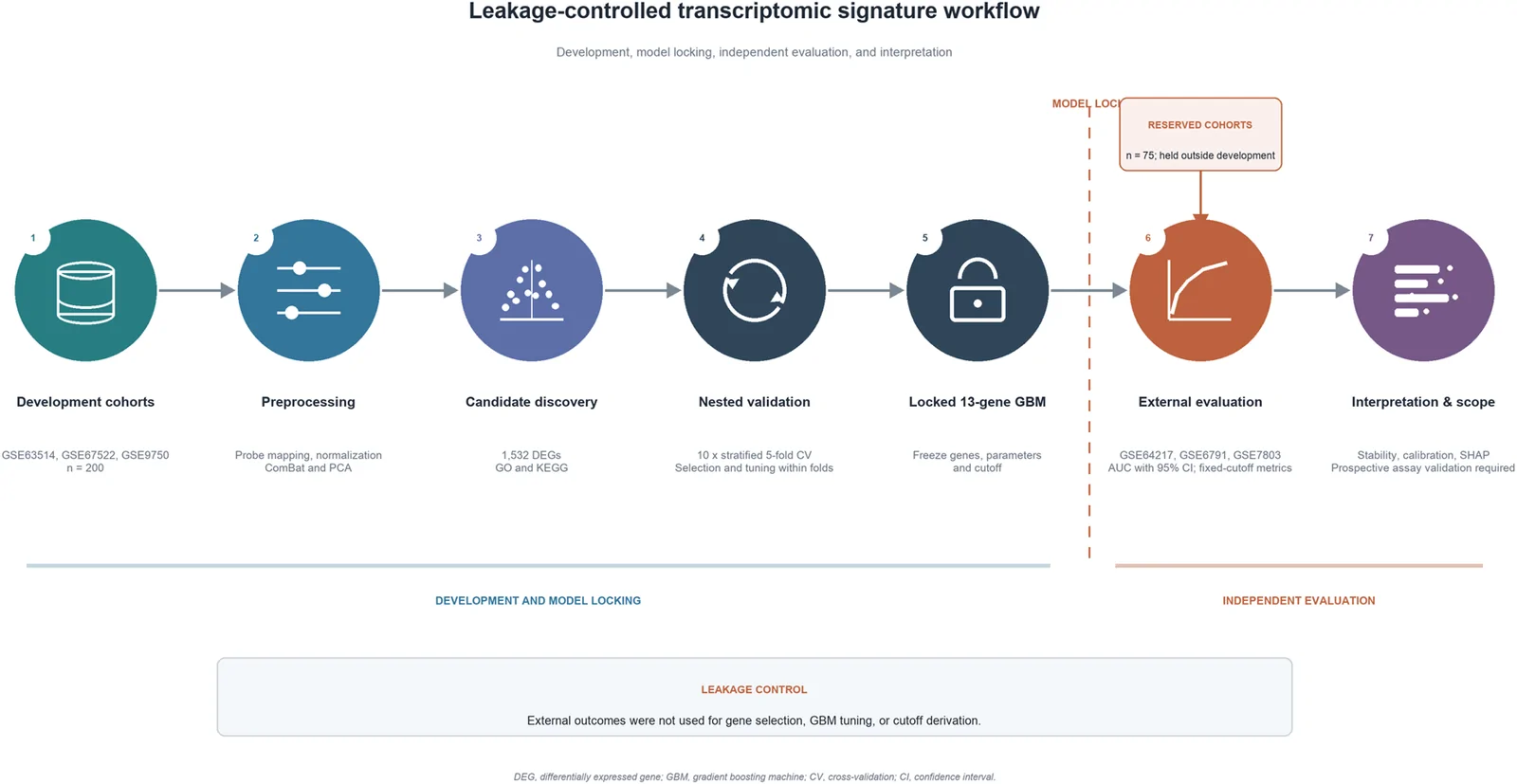
Development, validation, and limitation-aware evaluation workflow. The development pipeline comprised cohort integration, inherited batch adjustment, candidate-gene discovery, repeated nested supervised validation, and model locking. GSE64217, GSE6791, and GSE7803 were used only after the final model and cutoff had been locked. The workflow distinguishes supervised stages protected by resampling from the historical cohort-wide ComBat preprocessing, which is addressed explicitly as a limitation in the Methods and Discussion.

Preprocessing quality control showed strong GEO-series separation before ComBat and reduced separation afterwards. For the 500 most variable genes, the variance explained by PC1 plus PC2 fell from 0.658 before ComBat to 0.341 after ComBat, while the mean GEO-series silhouette across the first 10 PCs fell from 0.552 to −0.011 (Supplementary Table S6; Supplementary Figure S2C). These are descriptive processing indices, not evidence that all batch effects or biological confounding were removed.

Differential expression analysis tested 10,783 genes and identified 1,532 differentially expressed genes, including 811 upregulated and 721 downregulated genes under the prespecified limma and FDR criteria (Benjamini and Hochberg, 1995; Ritchie et al., 2015). Leading upregulated genes included MMP12, CDKN2A, APOC1, SPP1, and SYCP2, whereas CRNN, CRISP3, MAL, UPK1A, and SLURP1 were among the most downregulated. The PCA omits individual sample labels, and the heatmap is restricted to leading genes to improve readability (Figure 2).

**FIGURE 2:**
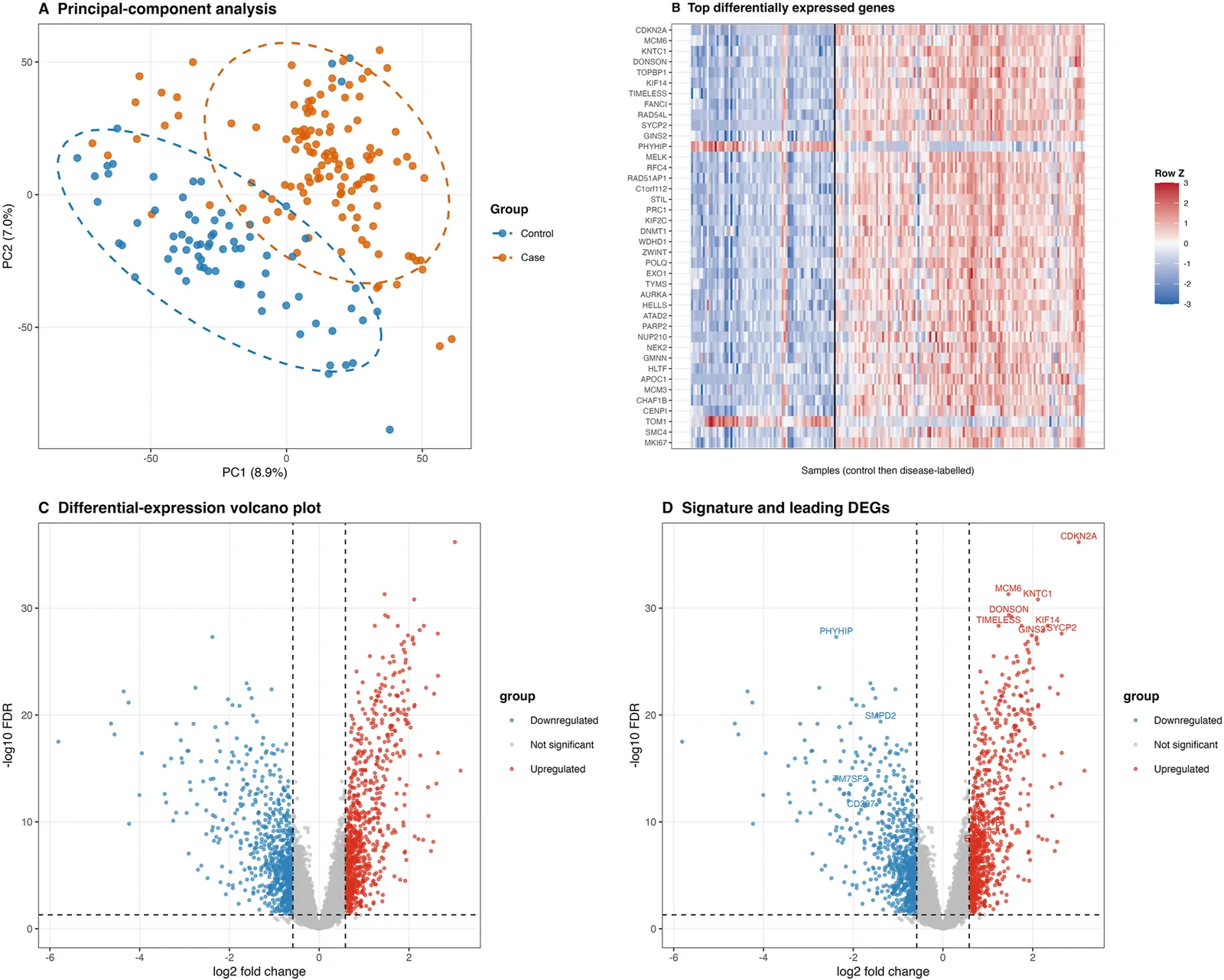
Differential-expression landscape in the 200-sample development cohort. **(A)** Principal-component analysis without individual sample labels. Ellipses show 95% group regions. **(B)** Row-standardized expression heatmap of 40 leading differentially expressed genes, with control samples followed by case samples. **(C)** Volcano plot using absolute log2 fold change >0.585 and FDR-adjusted P < 0.05. **(D)** Selected leading genes and signature genes are labelled. Red indicates upregulation, blue indicates downregulation, and gray indicates non-significance.

At the original 1.5-fold effect-size threshold, 1,532 genes met the FDR criterion. Tightening only the effect-size threshold to two-fold retained 559 genes (280 upregulated and 279 downregulated); four of the fixed 13 panel genes (AKR1B1, CD207, SMPD2, and TM7SF2) met this more stringent descriptive threshold (Supplementary Table S7). This calculation does not reselect the panel or estimate a two-fold-threshold classifier, and is included only to show how the descriptive DEG count changes with the screening threshold.

### Functional enrichment contextualized the candidate genes

Five KEGG pathways met the FDR criterion: steroid biosynthesis, sphingolipid metabolism, taurine and hypotaurine metabolism, other glycan degradation, and drug metabolism-cytochrome P450. Sphingolipid metabolism included SMPD2, HEXA, and NEU1. GO enrichment similarly highlighted sphingolipid metabolic process, ceramide metabolic process, membrane lipid metabolic process, lipid catabolic process, and sphingolipid catabolic process (Ashburner et al., 2000; Kanehisa and Goto, 2000; Yu et al., 2012; Gene Ontology Consortium, 2021; Kanehisa et al., 2021; Figure 3). These analyses provided biological annotation of the candidate genes only; they were not used to select the final classifier or to validate diagnostic performance.

**FIGURE 3:**
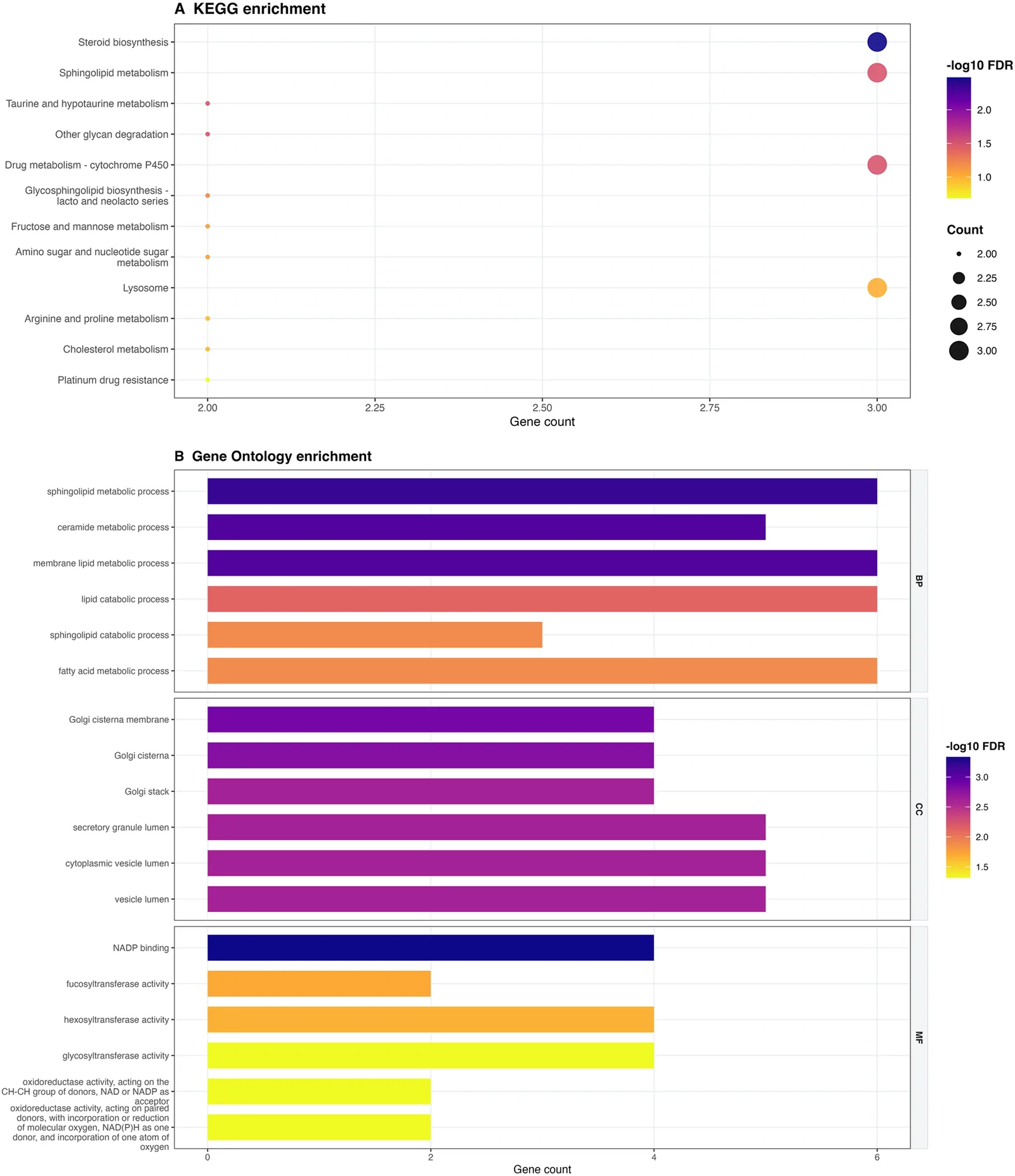
KEGG and Gene Ontology enrichment of candidate genes. **(A)** KEGG dot plot; point size represents gene count and color represents -log10 FDR. **(B)** GO biological process (BP), cellular component (CC), and molecular function (MF) results; bar length represents gene count and color represents -log10 FDR.

### Nested validation estimated internal discrimination and calibration

Repeated nested validation produced an aggregated out-of-fold AUC of 0.9161 (95% CI 0.8731–0.9591), below the original resubstitution estimate of approximately 0.99. This reduction was expected because every feature-selection and tuning step was repeated without access to the held-out outer fold (Ambroise and McLachlan, 2002; Varma and Simon, 2006; Steyerberg et al., 2010; Riley et al., 2020). Across the 10 repeats, the mean AUC was 0.9067 (SD 0.0091; range 0.8945–0.9210). At the out-of-fold Youden cutoff of 0.6136, sensitivity was 0.8898 (95% CI 0.8220–0.9384), specificity was 0.8082 (95% CI 0.6992–0.8910), and accuracy was 0.8600; PPV and NPV were 0.8898 and 0.8082, respectively. The Brier score was 0.1022, the calibration intercept was −0.1439, and the calibration slope was 1.0536 (Brier, 1950; Van Calster et al., 2019; Table 2 and Figure 4).

**TABLE 2:** Nested internal and locked external model performance.

Cohort	n	AUC (95% CI)	Sensitivity	Specificity	Accuracy	Brier
Repeated nested CV OOF	200	0.9161 (0.8731–0.9591)	0.8898	0.8082	0.8600	0.1022
GSE64217	6	1.0000 (n = 6; descriptive only)	1.0000	1.0000	1.0000	0.0304
GSE6791	28	0.9938 (0.9764–1.0000)	0.9000	1.0000	0.9286	0.0561
GSE7803	41	0.9167 (0.8299–1.0000)	0.7812	0.8889	0.8049	0.1306
GSE6791 + GSE7803	69	0.9389 (0.8832–0.9946)	0.8269	0.9412	0.8551	0.1004

AUC, confidence intervals are DeLong intervals. The fixed threshold was 0.6136. For GSE64217 (n = 6), the apparent perfect point estimates are descriptive only and no precise AUC, inference is made; exact 95% confidence intervals for sensitivity and specificity are 0.3976–1.0000 and 0.1581–1.0000, respectively.

**FIGURE 4:**
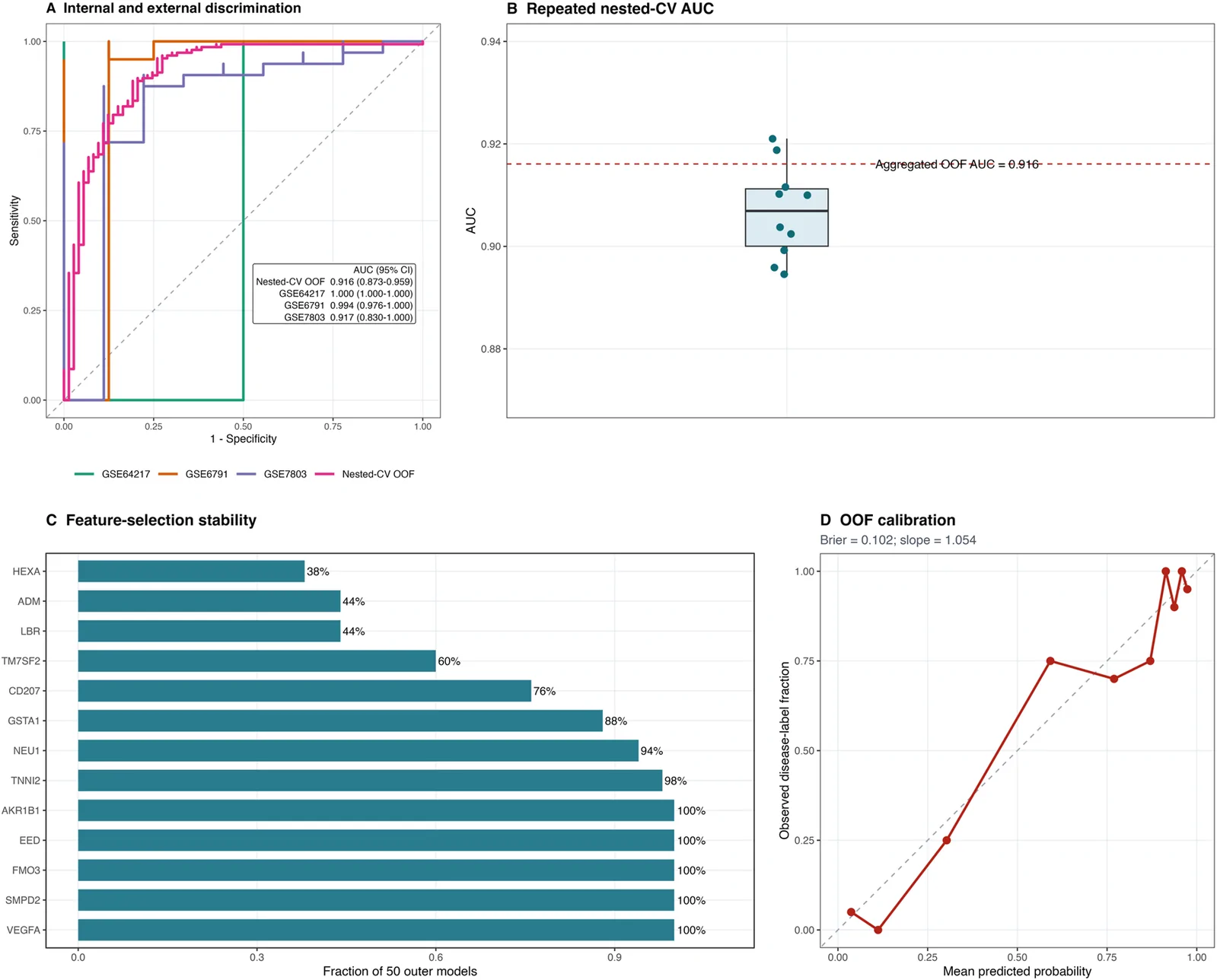
Nested-validation model performance and selection stability. **(A)** ROC curves for aggregated nested-cross-validation out-of-fold predictions and the three external cohorts, with DeLong 95% confidence intervals shown in the inset. The GSE64217 curve is based on only six samples. **(B)** Distribution of AUCs across 10 repeated nested-cross-validation runs. **(C)** Selection frequency of the 13 panel genes across 50 outer models. **(D)** Decile-based calibration of aggregated out-of-fold probabilities; Brier score and calibration slope are shown.

### Locked external evaluation showed cohort-dependent discrimination

Once the 13-gene GBM and cutoff had been locked, the cohort-wise standardized external AUC was 1.0000 in GSE64217, 0.9938 (95% CI 0.9764–1.0000) in GSE6791, and 0.9167 (95% CI 0.8299–1.0000) in GSE7803. At the fixed cutoff, sensitivity/specificity were 1.0000/1.0000, 0.9000/1.0000, and 0.7813/0.8889, respectively. The nominally perfect result in GSE64217 was not robust evidence of perfect diagnostic performance: exact 95% confidence intervals were 0.3976–1.0000 for sensitivity and 0.1581–1.0000 for specificity because only six samples were analyzed. When GSE64217 was excluded, the pooled GSE6791/GSE7803 external AUC was 0.9389 (95% CI 0.8832–0.9946), which is a more informative summary than the six-sample perfect estimate.

Applying frozen development scaling produced similar AUCs but slightly lower threshold-dependent performance in GSE7803 (AUC 0.9132; sensitivity 0.7500; specificity 0.8889), supporting the conclusion that platform and normalization choices remain relevant to deployment (Supplementary Table S3).

### Feature stability, *post hoc* simplification, and SHAP analysis

The outer models selected a median of 12 genes (range 8–15). SMPD2, VEGFA, AKR1B1, EED, and FMO3 were selected in all 50 outer models; TNNI2 was selected in 98%, NEU1 in 94%, GSTA1 in 88%, and CD207 in 76%. TM7SF2 was selected in 60%, ADM and LBR in 44%, and HEXA in 38% (Figure 4; Supplementary Table S4). The exact 13-gene composition was therefore less stable than the original single fit suggested, consistent with the known instability of high-dimensional variable selection (Guyon and Elisseeff, 2003; Steyerberg et al., 2010). These results identify a stable five-gene core and a less stable extended panel.

To test whether the stable five-gene core supported a materially different fixed-panel signal, we conducted a *post hoc* sensitivity analysis using SMPD2, VEGFA, AKR1B1, EED, and FMO3 versus the fixed 13-gene panel. On the inherited ComBat-adjusted matrix, 10 repeats of stratified five-fold outer validation with training-fold scaling and inner GBM tree selection gave an AUC of 0.9308 (95% CI 0.8950–0.9665) for the five-gene core and 0.9241 (95% CI 0.8835–0.9646) for the 13-gene panel; the paired DeLong comparison was not significant (P = 0.3895). Calibration summaries were similarly modest (13-gene ECE 0.0469, ICI 0.0228; five-gene ECE 0.0630, ICI 0.0097). This result does not establish equivalence, superiority, or a replacement model because the core was defined from the same development data and the comparison was *post hoc* (Supplementary Table S5; Supplementary Figure S2A).

All 13 genes were differentially expressed in the development cohort (Table 3). SMPD2 was downregulated (logFC −1.3905; FDR-adjusted P = 4.10 x 10^-20) and had the highest single-gene AUC, 0.8576 (95% CI 0.7978–0.9175). TM7SF2 ranked second by single-gene AUC (0.8277), followed by AKR1B1 (0.7938). Replacing the crowded 13-curve ROC panel with a ranked AUC plot made uncertainty directly visible (Figure 5).

**TABLE 3:** Differential expression, single-gene discrimination, SHAP importance, and selection stability of the 13-gene panel.

Gene	logFC	FDR	AUC (95% CI)	Mean |SHAP|	Outer selection	Function/cancer-relevant context
SMPD2	−1.3905	4.10e-20	0.8576 (0.7978–0.9175)	0.1402	100%	Neutral sphingomyelinase; sphingomyelin/ceramide metabolism
VEGFA	0.9498	3.23e-09	0.7707 (0.7059–0.8355)	0.0771	100%	Angiogenesis and hypoxia-associated signaling
AKR1B1	1.0237	4.33e-10	0.7938 (0.7291–0.8585)	0.0540	100%	Aldo-keto reductase; oxidative and carbonyl-stress response
EED	0.6717	1.29e-08	0.7651 (0.6967–0.8336)	0.0632	100%	PRC2 component; epigenetic transcriptional repression
TM7SF2	−2.0608	3.39e-14	0.8277 (0.7633–0.8922)	0.0156	60%	Sterol and cholesterol biosynthesis-related enzyme
HEXA	0.6070	4.51e-09	0.7701 (0.6985–0.8416)	0.0410	38%	Lysosomal hexosaminidase; glycolipid degradation
FMO3	0.7065	1.07e-02	0.5592 (0.4748–0.6435)	0.0242	100%	Flavin monooxygenase; xenobiotic/drug metabolism
GSTA1	−0.7169	5.05e-05	0.7523 (0.6832–0.8214)	0.0294	88%	Glutathione transferase; detoxification and oxidative stress
NEU1	0.7313	1.92e-06	0.7100 (0.6326–0.7874)	0.0274	94%	Lysosomal sialidase; glycoprotein and ganglioside metabolism
ADM	0.7029	4.53e-04	0.6536 (0.5759–0.7313)	0.0274	44%	Adrenomedullin; stress, hypoxia, and angiogenic signaling
TNNI2	0.7217	4.38e-04	0.6529 (0.5763–0.7295)	0.0192	98%	Troponin I isoform; differentiation-associated candidate marker
CD207	−1.8249	7.38e-12	0.7747 (0.7038–0.8455)	0.0084	76%	Langerin; immune-cell and antigen-presentation marker
LBR	0.6178	5.26e-09	0.7584 (0.6881–0.8287)	0.0090	44%	Nuclear envelope/sterol-reductase-related protein

FDR, false-discovery-rate adjusted P value; SHAP, Shapley additive explanation. Outer selection is the percentage of 50 nested-cross-validation outer models selecting each gene. The function/cancer-relevant context column is a descriptive biological annotation and does not establish a causal role in cervical neoplasia.

**FIGURE 5:**
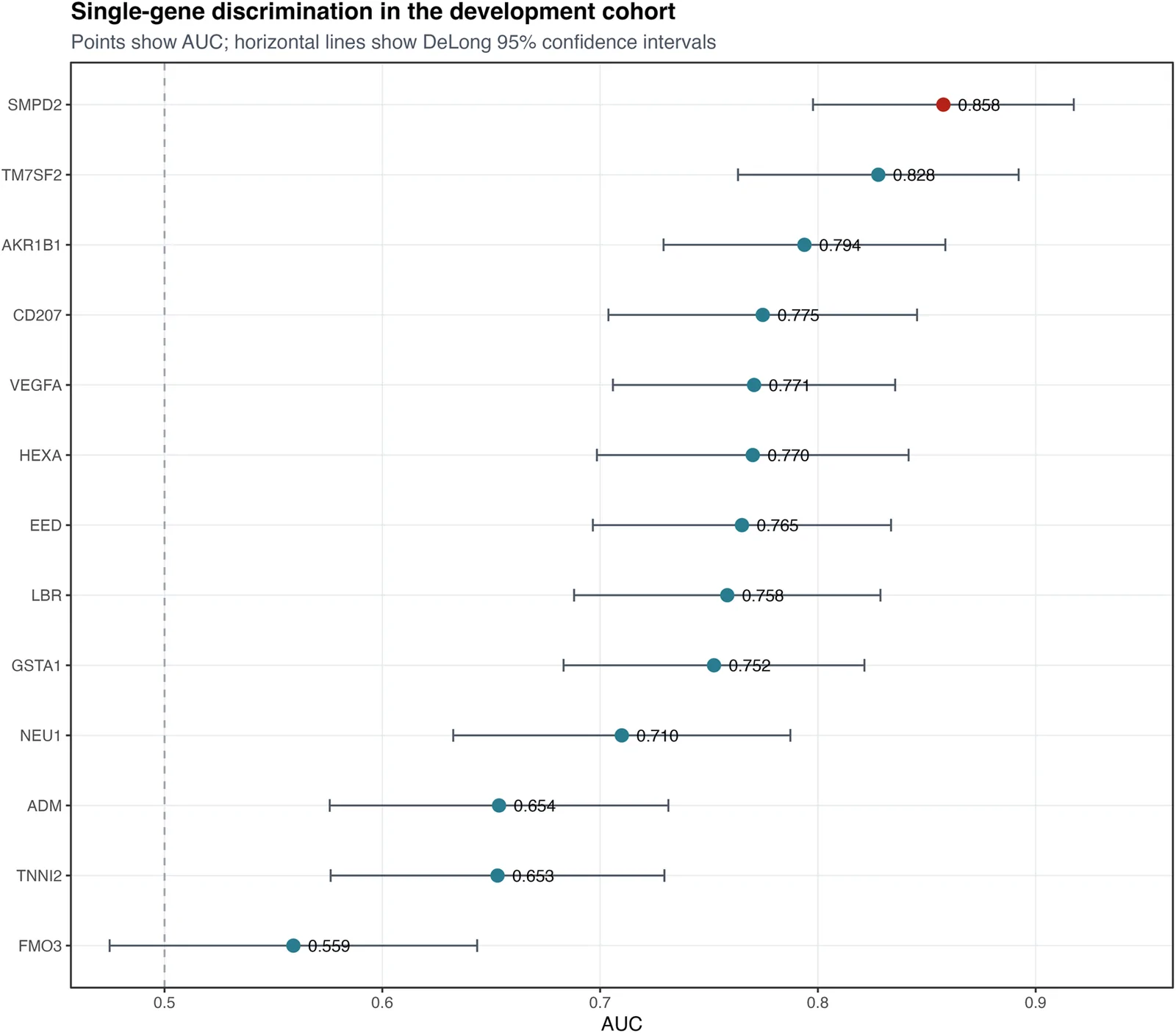
Single-gene discrimination in the development cohort. Points show direction-standardized AUCs and horizontal lines show DeLong 95% confidence intervals for the 13 signature genes. SMPD2 is highlighted.

Kernel SHAP analysis of the locked GBM ranked SMPD2 first (mean absolute SHAP 0.1402), followed by VEGFA (0.0771), EED (0.0632), AKR1B1 (0.0540), and HEXA (0.0410). The revised SHAP figure retains the global importance and distribution panels and removes the small, overlapping dependence panels from the main figure (Figure 6).

**FIGURE 6:**
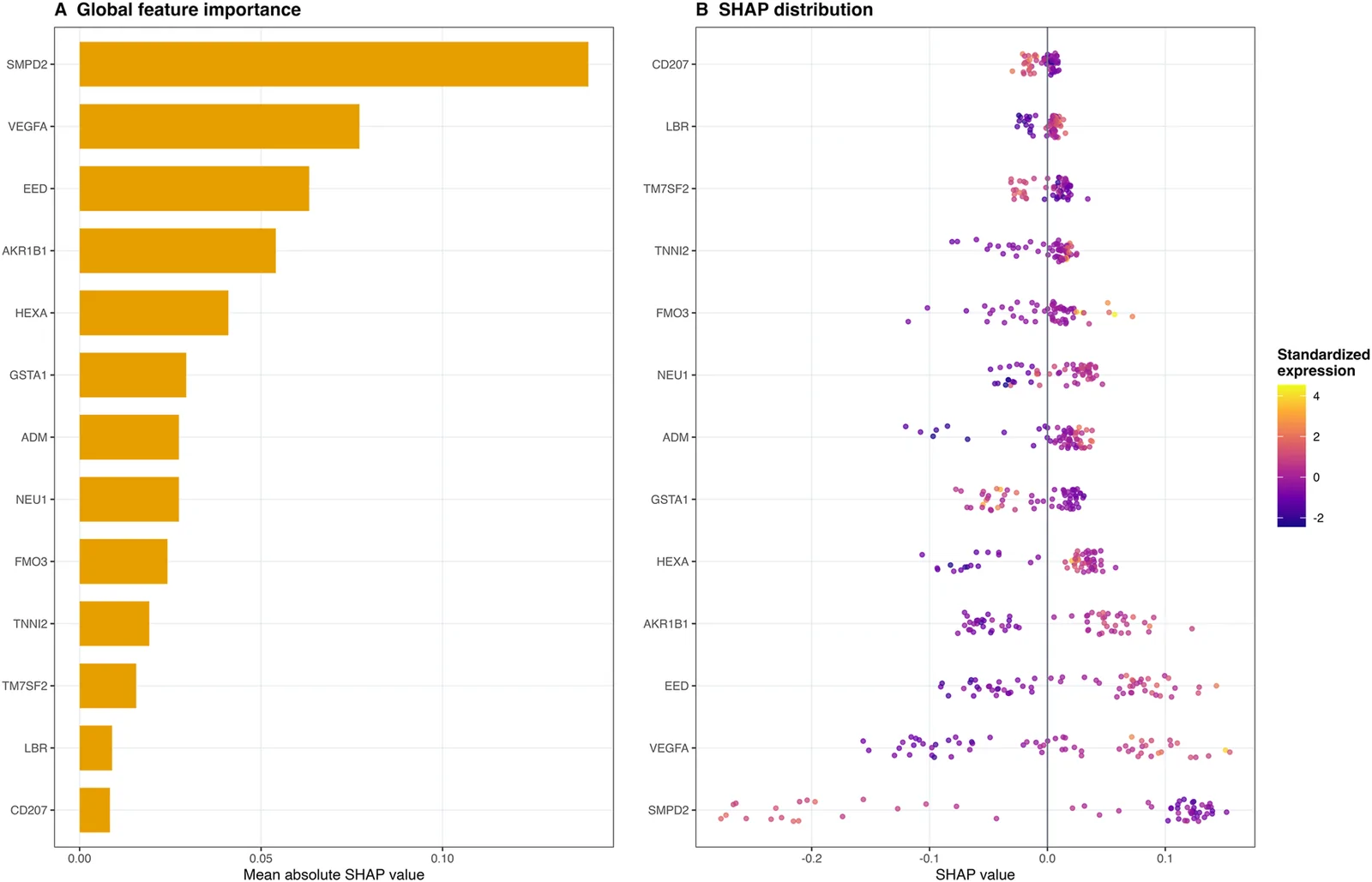
SHAP interpretation of the locked 13-gene GBM. **(A)** Mean absolute SHAP value across a seed-fixed stratified subset of 60 development samples. **(B)** SHAP value distribution; color represents standardized gene expression. SHAP values are descriptive and do not establish causality.

## Discussion

The principal contribution of this reanalysis is not a clinically deployable diagnostic test, but a transparent estimate of what remains after correcting selected sources of optimism in a retrospective GEO microarray workflow. The 13-gene glmBoost-GBM candidate classifier retained discrimination after nested supervised validation and model-locked external evaluation in the inherited cohort set. This evidence is stronger than a resubstitution estimate because feature selection and GBM tuning were repeated within outer-training splits, external outcomes were withheld from the reconstructed model-selection stages, and the decision threshold was fixed from development out-of-fold predictions before external evaluation. It remains incomplete because the cohort-wide ComBat adjustment preceded resampling and cannot be retrospectively represented as fully foldwise preprocessing.

The most important unresolved limitation is the absence of independent biological validation. The study uses only public retrospective microarray datasets and does not validate the signature in TCGA-CESC, GTEx-normal tissue comparisons, RNA-seq cohorts, qPCR assays, independent patient samples, tissue microarrays, or immunohistochemistry. We did not add a new TCGA-CESC-versus-normal comparison because tissue-source, platform, and control-origin differences would create a different biological and technical contrast rather than a clean validation of the locked GEO microarray classifier. The revised manuscript therefore frames the work as a strictly computational prioritization and validation-boundary study. Prospective assay-level validation is required before the panel can be considered for clinical deployment.

Dataset heterogeneity further constrains the modeled endpoint. The source series differ in platform, specimen acquisition, microdissection, HPV composition, histology, and disease spectrum. Several processed case groups combine CIN or HSIL with invasive cancer, and the source GSE9750 series includes cell-line material. The classifier may therefore capture a broad cervical neoplastic or epithelial state rather than an invasive-cancer-specific signal. This distinction creates possible spectrum bias, class ambiguity, and label noise, and it means that performance cannot be transferred automatically to screening, triage, colposcopy referral, or histology-specific diagnostic settings. Future studies should prospectively define clinical categories, distinguish precursor lesions from invasive disease, apply a prespecified sampling frame, and report performance by specimen type, histology, stage, HPV status, and intended clinical use.

SMPD2 provides a biologically interpretable link between the statistical model and lipid metabolism, although causality is not established. SMPD2 encodes neutral sphingomyelinase 1, whose catalytic architecture has recently been resolved (Yi et al., 2023). Ceramide and sphingomyelin pathways regulate membrane organization, cellular stress, proliferation, apoptosis, and treatment response (Ogretmen and Hannun, 2004; Hannun and Obeid, 2018). These processes are relevant to HPV-driven epithelial transformation because viral oncoproteins remodel host cell-cycle control, apoptosis, immune recognition, and tissue microenvironmental programs during progression from infection to precancer and cancer (Doorbar et al., 2012; den Boon et al., 2015; Schiffman et al., 2016; Cancer Genome Atlas Research Network, 2017). In this study, SMPD2 was downregulated, belonged to the FDR-significant sphingolipid metabolism pathway with HEXA and NEU1, had the highest single-gene AUC, was selected in all outer folds, and ranked first by SHAP importance. This convergence supports SMPD2 as a candidate marker connecting the classifier to sphingolipid and ceramide biology, but functional experiments are needed to test whether SMPD2 participates in HPV-mediated lipid remodeling, apoptosis resistance, or immune escape in cervical neoplasia.

### Potential clinical translation

The 13-gene classifier should be viewed as a research-grade candidate computational model, not as a replacement for HPV testing, cytology, colposcopy, or histopathology. The current analysis does not validate any clinical assay platform for the panel, compare it directly with HPV-associated biomarkers or published transcriptomic signatures, or demonstrate incremental clinical utility. The *post hoc* out-of-fold decision-analytic display is included only to show the form of a future utility analysis; because the data lack a defined clinical decision, comparator, action threshold, and prospective outcome, it cannot establish net clinical benefit. If the panel is pursued in future work, assay development should begin with a specified specimen type, preanalytical handling protocol, RNA-quality threshold, single-sample normalization strategy, locked coefficients and cutoff, analytical sensitivity and specificity, inter-laboratory reproducibility, and blinded clinical validation in a defined screening or triage context. Most importantly, the present cohort-wise z-standardization cannot score a single future patient and must be replaced by a frozen or reference-based normalization procedure before prospective clinical testing.

### Strengths and limitations

Strengths include the explicit code audit, separation of internal and external validation, repeated nested cross-validation with feature selection and tuning inside the outer loop, reporting of threshold-dependent metrics and calibration, and open audit outputs. The reporting also makes the inherited cohort-selection process, label heterogeneity, feature-instability signal, and assay-development gap explicit rather than presenting the model as a clinically deployable diagnostic test.

Several limitations remain. The study is retrospective, strictly computational, and based on public microarray data. A formal GEO search log was not available, so dataset selection may be incomplete or biased. Disease labels and specimen composition are heterogeneous, and some cohorts include precursor lesions or cell-line material. GSE64217 is too small for precise diagnostic estimates and should not be used to imply perfect performance. Cohort-wise standardization is transductive and cannot support single-patient deployment. The inherited ComBat transformation was estimated before reconstructed resampling; the nested analysis therefore does not establish fully foldwise preprocessing validation. The fixed panel was inherited from the development-stage analysis rather than selected by a prospectively registered protocol, and several genes were not consistently selected across outer folds. The new five-versus-13-gene comparison, ECE/ICI estimates, and decision-analytic display are *post hoc* sensitivity analyses rather than independent confirmation. No external biomarker benchmark, TCGA/RNA-seq replication, qPCR validation, tissue-level validation, GSEA, pathway-activity scoring, clinical impact curve, or net reclassification analysis was performed. SHAP values are descriptive, and no functional experiment establishes a causal role for SMPD2. Prospective analytical and clinical validation is required before any claim of clinical utility.

## Conclusion

This transparent reanalysis of six inherited GEO cohorts shows that a glmBoost-GBM candidate classifier retains a measurable cervical neoplasia-related transcriptomic signal after a more rigorous reconstruction of supervised model evaluation. The 13-gene candidate classifier achieved an aggregated nested-cross-validation AUC of 0.9161 and high but cohort-dependent retrospective external discrimination, while a five-gene core was stable across outer fits and did not differ significantly from the fixed 13-gene panel in a *post hoc* sensitivity comparison. SMPD2 emerged as the most stable and interpretable individual feature. The findings support computational prioritization and hypothesis-driven evaluation of a cervical neoplastic-spectrum signal, but they do not establish biological validation, superiority to HPV-based or published transcriptomic biomarkers, clinical utility, fully foldwise preprocessing, or a deployable diagnostic test. Clinical translation requires prospectively selected homogeneous cohorts, independent biological and assay validation, fixed single-sample normalization, locked model parameters, and a predefined clinical decision context.

## Data Availability

The analyzed datasets are publicly available in the NCBI Gene Expression Omnibus under accession numbers GSE63514, GSE67522, GSE9750, GSE64217, GSE6791, and GSE7803. Derived analysis outputs are provided in the Supplementary Material. Further inquiries can be directed to the corresponding author.

## Supplementary material

SUPPLEMENTARY FIGURE S1 Descriptive principal-component analysis before and after ComBat batch adjustment of the three development GEO matrices. **(A)** Combined development matrix before ComBat, colored by GEO series. **(B)** Matrix after ComBat, colored by GEO series. The figure is a descriptive preprocessing quality-control display; quantitative indices and an updated before/after PCA display are reported in Supplementary Figure S2C and Supplementary Table S6. Neither display proves preservation of biological signal or validates model performance.

SUPPLEMENTARY FIGURE S2 Post hoc stability, decision-analytic, and batch-quality-control sensitivity analyses. **(A)** Repeated-CV out-of-fold ROC curves for the fixed 13-gene panel and the five-gene core. The paired DeLong test was not significant (P = 0.3895). **(B)** Exploratory decision-analytic curves derived from the same out-of-fold probabilities. Because no prospective clinical decision, comparator, or outcome was defined, this panel does not establish clinical utility. **(C)** PCA before and after ComBat colored by GEO series; quantitative PC variance and silhouette indices are reported in Supplementary Table S6. The inherited cohort-wide ComBat step remains a stated preprocessing limitation.
