# Supplementary material for: A transparent 13-gene transcriptomic candidate signature for cervical neoplastic spectrum across GEO cohorts
Source: Front Genet. 2026 Oct 30;17:1875050. doi: 10.3389/fgene.2026.1875050 (PMC13759488; doi:10.3389/fgene.2026.1875050)
Supplement: Supplementary file 1 [file Table1.docx]

Supplementary Materials

Manuscript 1875050 | Revised submission

These tables document the original model-screening output and the corrected leakage-controlled analysis. The original 121-model matrix is retained for transparency but was not used as an unbiased external-validation comparison in the revised analysis.

# Supplementary Table S1. Original 121-model AUC matrix (audit record).

| **Method** | **Train** | **GSE64217** | **GSE6791** | **GSE7803** | **Original mean** |
| --- | --- | --- | --- | --- | --- |
| Lasso+Stepglm[both] | 0.949196418940783 | 1 | 0.925 | 0.861111111111111 | 0.9338 |
| SVM | 0.922608132887499 | 1 | 1 | 0.9375 | 0.9650 |
| glmBoost+SVM | 0.92158343220796 | 1 | 0.95 | 0.850694444444444 | 0.9306 |
| Ridge | 0.947362744040557 | 1 | 1 | 0.944444444444444 | 0.9730 |
| Lasso+SVM | 0.925520440081976 | 1 | 0.975 | 0.850694444444444 | 0.9378 |
| glmBoost+Ridge | 0.947578470499407 | 1 | 1 | 0.913194444444444 | 0.9652 |
| Enet[alpha=0.1] | 0.943587531010678 | 1 | 1 | 0.9375 | 0.9703 |
| glmBoost+Enet[alpha=0.1] | 0.949627871858483 | 1 | 1 | 0.90625 | 0.9640 |
| Enet[alpha=0.2] | 0.944558300075504 | 1 | 1 | 0.927083333333333 | 0.9679 |
| Enet[alpha=0.3] | 0.949520008629058 | 1 | 1 | 0.913194444444444 | 0.9657 |
| glmBoost+Enet[alpha=0.3] | 0.949304282170208 | 1 | 1 | 0.90625 | 0.9639 |
| glmBoost+Enet[alpha=0.2] | 0.949088555711358 | 1 | 1 | 0.909722222222222 | 0.9647 |
| Enet[alpha=0.4] | 0.948872829252508 | 1 | 1 | 0.913194444444444 | 0.9655 |
| glmBoost+Enet[alpha=0.4] | 0.949520008629058 | 1 | 1 | 0.899305555555556 | 0.9622 |
| Lasso+glmBoost | 0.943371804551828 | 1 | 1 | 0.888888888888889 | 0.9581 |
| Enet[alpha=0.5] | 0.948441376334808 | 1 | 1 | 0.913194444444444 | 0.9654 |
| glmBoost | 0.943479667781253 | 1 | 1 | 0.902777777777778 | 0.9616 |
| glmBoost+Enet[alpha=0.5] | 0.950167188005609 | 1 | 1 | 0.899305555555556 | 0.9624 |
| Enet[alpha=0.6] | 0.948441376334808 | 1 | 1 | 0.90625 | 0.9637 |
| glmBoost+Enet[alpha=0.6] | 0.949196418940783 | 1 | 1 | 0.899305555555556 | 0.9621 |
| glmBoost+Enet[alpha=0.7] | 0.948980692481933 | 1 | 1 | 0.899305555555556 | 0.9621 |
| glmBoost+Enet[alpha=0.8] | 0.949951461546759 | 1 | 1 | 0.899305555555556 | 0.9623 |
| Enet[alpha=0.8] | 0.947902060187682 | 1 | 1 | 0.899305555555556 | 0.9618 |
| Enet[alpha=0.9] | 0.947362744040557 | 1 | 1 | 0.892361111111111 | 0.9599 |
| Lasso | 0.947578470499407 | 1 | 1 | 0.888888888888889 | 0.9591 |
| Enet[alpha=0.7] | 0.948225649875957 | 1 | 1 | 0.899305555555556 | 0.9619 |
| glmBoost+Enet[alpha=0.9] | 0.948764966023083 | 1 | 1 | 0.899305555555556 | 0.9620 |
| glmBoost+Lasso | 0.948980692481933 | 1 | 1 | 0.892361111111111 | 0.9603 |
| Lasso+plsRglm | 0.944126847157804 | 1 | 1 | 0.878472222222222 | 0.9556 |
| glmBoost+plsRglm | 0.937655053392298 | 1 | 0.99375 | 0.909722222222222 | 0.9603 |
| glmBoost+Stepglm[forward] | 0.951785136446985 | 1 | 0.9875 | 0.895833333333333 | 0.9588 |
| Lasso+Stepglm[forward] | 0.95167727321756 | 1 | 1 | 0.892361111111111 | 0.9610 |
| RF+SVM | 0.922608132887499 | 1 | 1 | 0.881944444444444 | 0.9511 |
| Stepglm[forward] | 0.968611800237299 | 1 | 0.90625 | 0.829861111111111 | 0.9262 |
| plsRglm | 0.940351634127926 | 1 | 0.99375 | 0.927083333333333 | 0.9653 |
| RF+Ridge | 0.940351634127926 | 1 | 1 | 0.9375 | 0.9695 |
| RF+Enet[alpha=0.1] | 0.938410095998274 | 1 | 1 | 0.9375 | 0.9690 |
| RF+plsRglm | 0.932693344838744 | 1 | 1 | 0.913194444444444 | 0.9615 |
| RF+Stepglm[forward] | 0.950814367382159 | 1 | 0.98125 | 0.871527777777778 | 0.9509 |
| RF+Enet[alpha=0.2] | 0.939488728292525 | 1 | 1 | 0.927083333333333 | 0.9666 |
| RF+Enet[alpha=0.3] | 0.9398123179808 | 1 | 1 | 0.923611111111111 | 0.9659 |
| RF+Enet[alpha=0.6] | 0.940998813504476 | 1 | 1 | 0.913194444444444 | 0.9635 |
| RF+Lasso | 0.940567360586776 | 1 | 0.98125 | 0.895833333333333 | 0.9544 |
| RF+Enet[alpha=0.7] | 0.940890950275051 | 1 | 1 | 0.913194444444444 | 0.9635 |
| RF+Enet[alpha=0.5] | 0.942077445798727 | 1 | 1 | 0.913194444444444 | 0.9638 |
| RF+glmBoost | 0.937007874015748 | 1 | 1 | 0.902777777777778 | 0.9599 |
| RF+Enet[alpha=0.9] | 0.941322403192752 | 1 | 0.9875 | 0.90625 | 0.9588 |
| RF+Enet[alpha=0.4] | 0.941861719339877 | 1 | 1 | 0.913194444444444 | 0.9638 |
| RF+Enet[alpha=0.8] | 0.940890950275051 | 1 | 0.99375 | 0.909722222222222 | 0.9611 |
| RF+Stepglm[both] | 0.9398123179808 | 1 | 0.95 | 0.868055555555556 | 0.9395 |
| RF+Stepglm[backward] | 0.9398123179808 | 1 | 0.95 | 0.868055555555556 | 0.9395 |
| Stepglm[both]+Ridge | 0.950382914464459 | 1 | 0.975 | 0.868055555555556 | 0.9484 |
| Stepglm[backward]+Ridge | 0.950382914464459 | 1 | 0.975 | 0.868055555555556 | 0.9484 |
| Stepglm[both]+plsRglm | 0.952648042282386 | 1 | 0.9375 | 0.84375 | 0.9335 |
| Stepglm[backward]+plsRglm | 0.952648042282386 | 1 | 0.9375 | 0.84375 | 0.9335 |
| Stepglm[both]+Enet[alpha=0.9] | 0.945852658828605 | 1 | 0.96875 | 0.868055555555556 | 0.9457 |
| Stepglm[backward]+Enet[alpha=0.9] | 0.94574479559918 | 1 | 0.9625 | 0.868055555555556 | 0.9441 |
| Stepglm[both]+Enet[alpha=0.1] | 0.953618811347212 | 1 | 0.95625 | 0.864583333333333 | 0.9436 |
| Stepglm[backward]+Enet[alpha=0.1] | 0.956099665623989 | 1 | 0.95 | 0.854166666666667 | 0.9401 |
| Stepglm[both]+Enet[alpha=0.8] | 0.958580519900766 | 1 | 0.94375 | 0.84375 | 0.9365 |
| Stepglm[backward]+Enet[alpha=0.8] | 0.957501887606515 | 1 | 0.95 | 0.847222222222222 | 0.9387 |
| Stepglm[both]+Enet[alpha=0.2] | 0.954805306870888 | 1 | 0.95625 | 0.864583333333333 | 0.9439 |
| Stepglm[backward]+Enet[alpha=0.2] | 0.956638981771114 | 1 | 0.95 | 0.850694444444444 | 0.9393 |
| Stepglm[both]+Lasso | 0.94552906914033 | 1 | 0.9625 | 0.868055555555556 | 0.9440 |
| Stepglm[backward]+Lasso | 0.946284111746306 | 1 | 0.9625 | 0.868055555555556 | 0.9442 |
| Stepglm[both]+Enet[alpha=0.6] | 0.947254880811132 | 1 | 0.975 | 0.871527777777778 | 0.9484 |
| Stepglm[backward]+Enet[alpha=0.6] | 0.953726674576637 | 1 | 0.95 | 0.864583333333333 | 0.9421 |
| glmBoost+GBM | 0.990508035810592 | 1 | 0.98125 | 0.913194444444444 | 0.9712 |
| Stepglm[both]+Enet[alpha=0.7] | 0.946823427893431 | 1 | 0.975 | 0.871527777777778 | 0.9483 |
| Stepglm[backward]+Enet[alpha=0.7] | 0.947254880811132 | 1 | 0.975 | 0.871527777777778 | 0.9484 |
| Lasso+Stepglm[backward] | 0.949196418940783 | 1 | 0.925 | 0.861111111111111 | 0.9338 |
| Stepglm[both] | 0.959551288965592 | 1 | 0.91875 | 0.822916666666667 | 0.9253 |
| Stepglm[backward] | 0.959551288965592 | 1 | 0.91875 | 0.822916666666667 | 0.9253 |
| glmBoost+Stepglm[both] | 0.949196418940783 | 1 | 0.925 | 0.861111111111111 | 0.9338 |
| glmBoost+Stepglm[backward] | 0.949196418940783 | 1 | 0.925 | 0.861111111111111 | 0.9338 |
| Stepglm[both]+Enet[alpha=0.4] | 0.955991802394564 | 1 | 0.95 | 0.857638888888889 | 0.9409 |
| Stepglm[backward]+Enet[alpha=0.4] | 0.951785136446985 | 1 | 0.9625 | 0.868055555555556 | 0.9456 |
| Stepglm[both]+Enet[alpha=0.3] | 0.949304282170208 | 1 | 0.975 | 0.871527777777778 | 0.9490 |
| Stepglm[backward]+Enet[alpha=0.3] | 0.95717829791824 | 1 | 0.95 | 0.847222222222222 | 0.9386 |
| Stepglm[both]+glmBoost | 0.941969582569302 | 1 | 0.9625 | 0.868055555555556 | 0.9431 |
| Stepglm[backward]+glmBoost | 0.941969582569302 | 1 | 0.9625 | 0.868055555555556 | 0.9431 |
| Stepglm[both]+Enet[alpha=0.5] | 0.954481717182612 | 1 | 0.95 | 0.864583333333333 | 0.9423 |
| Stepglm[backward]+Enet[alpha=0.5] | 0.947578470499407 | 1 | 0.98125 | 0.871527777777778 | 0.9501 |
| glmBoost+RF | 0.996871966346672 | 1 | 0.99375 | 0.954861111111111 | 0.9864 |
| RF | 0.997087692805523 | 1 | 1 | 0.975694444444444 | 0.9932 |
| Lasso+GBM | 0.990076582892892 | 1 | 0.9875 | 0.885416666666667 | 0.9657 |
| RF+GBM | 0.996871966346672 | 1 | 1 | 0.892361111111111 | 0.9723 |
| GBM | 0.997195556034948 | 1 | 1 | 0.90625 | 0.9759 |
| Stepglm[both]+SVM | 0.928432747276454 | 1 | 0.925 | 0.763888888888889 | 0.9043 |
| Stepglm[backward]+SVM | 0.928432747276454 | 1 | 0.925 | 0.763888888888889 | 0.9043 |
| Lasso+RF | 0.996440513428972 | 1 | 0.99375 | 0.923611111111111 | 0.9785 |
| Stepglm[both]+GBM | 0.985977780174738 | 1 | 0.975 | 0.850694444444444 | 0.9529 |
| Stepglm[backward]+GBM | 0.988566497680941 | 1 | 0.96875 | 0.850694444444444 | 0.9520 |
| Stepglm[both]+RF | 0.996979829576098 | 1 | 0.9875 | 0.878472222222222 | 0.9657 |
| LDA | 0.947254880811132 | 1 | 1 | 0.961805555555556 | 0.9773 |
| glmBoost+LDA | 0.943048214863553 | 1 | 1 | 0.920138888888889 | 0.9658 |
| RF+LDA | 0.938086506309999 | 1 | 1 | 0.934027777777778 | 0.9680 |
| Stepglm[both]+LDA | 0.94488188976378 | 1 | 0.975 | 0.857638888888889 | 0.9444 |
| Stepglm[backward]+LDA | 0.94488188976378 | 1 | 0.975 | 0.857638888888889 | 0.9444 |
| Lasso+LDA | 0.944666163304929 | 1 | 1 | 0.913194444444444 | 0.9645 |
| Stepglm[backward]+RF | 0.996764103117247 | 1 | 0.99375 | 0.878472222222222 | 0.9672 |
| NaiveBayes | 0.906914033006148 | 1 | 1 | 0.996527777777778 | 0.9759 |
| Lasso+NaiveBayes | 0.942077445798727 | 1 | 1 | 0.9375 | 0.9699 |
| glmBoost+NaiveBayes | 0.935821378492072 | 1 | 1 | 0.982638888888889 | 0.9796 |
| RF+NaiveBayes | 0.917592492719232 | 1 | 1 | 0.996527777777778 | 0.9785 |
| Stepglm[both]+NaiveBayes | 0.931075396397368 | 1 | 1 | 0.9375 | 0.9671 |
| Stepglm[backward]+NaiveBayes | 0.931075396397368 | 1 | 1 | 0.9375 | 0.9671 |
| Stepglm[both]+RF+NaiveBayes | 0.997087692805523 | 1 | 0.9875 | 0.875 | 0.9649 |
| Lasso+GBM+RF | 0.992233847481394 | 1 | 0.9875 | 0.881944444444444 | 0.9654 |
| Stepglm[both]+Enet[alpha=0.8]+XGBoost | 0.946931291122856 | 1 | 0.975 | 0.871527777777778 | 0.9484 |
| Stepglm[backward]+Enet[alpha=0.8]+glmBoost | 0.946931291122856 | 1 | 0.975 | 0.871527777777778 | 0.9484 |
| Stepglm[both]+Enet[alpha=0.2]+GBM | 0.950490777693884 | 1 | 0.975 | 0.871527777777778 | 0.9493 |
| Stepglm[backward]+Enet[alpha=0.2]+XGBoost | 0.954805306870888 | 1 | 0.95625 | 0.864583333333333 | 0.9439 |
| Stepglm[both]+Lasso+GBM | 0.94552906914033 | 1 | 0.9625 | 0.868055555555556 | 0.9440 |
| Stepglm[backward]+Lasso+RF | 0.946284111746306 | 1 | 0.9625 | 0.868055555555556 | 0.9442 |
| Stepglm[both]+Enet[alpha=0.6]+GBM | 0.947254880811132 | 1 | 0.975 | 0.871527777777778 | 0.9484 |
| Stepglm[backward]+Enet[alpha=0.6]+GBM | 0.957070434688815 | 1 | 0.95 | 0.847222222222222 | 0.9386 |
| glmBoost+GBM+Lasso | 0.990723762269442 | 1 | 0.9875 | 0.923611111111111 | 0.9755 |
| Stepglm[both]+Enet[alpha=0.7]+GBM | 0.956531118541689 | 1 | 0.95 | 0.850694444444444 | 0.9393 |
| Stepglm[backward]+Enet[alpha=0.7]+NaiveBayes | 0.956531118541689 | 1 | 0.95 | 0.850694444444444 | 0.9393 |
| Lasso+Stepglm[backward]+Enet[alpha=0.5] | 0.949196418940783 | 1 | 0.925 | 0.861111111111111 | 0.9338 |

# Supplementary Table S2. Corrected glmBoost-GBM analysis parameters.

| **Item** | **Value** |
| --- | --- |
| Model | Development-stage glmBoost feature selection followed by locked GBM classification |
| Panel size | 13 |
| Panel genes | SMPD2, VEGFA, AKR1B1, EED, TM7SF2, HEXA, FMO3, GSTA1, NEU1, ADM, TNNI2, CD207, LBR |
| Random seed | 123 |
| Outer validation | 10 repeats of stratified 5-fold cross-validation |
| Inner tuning | 5-fold cross-validation within each outer-training split |
| GBM candidate trees | 10000 |
| GBM selected trees | 4192 |
| Interaction depth | 3 |
| Minimum terminal-node observations | 10 |
| Shrinkage | 0.001 |
| Bag fraction | 0.5 |
| Decision threshold | 0.613617 (Youden index from aggregated OOF predictions) |

# Supplementary Table S3. External-normalization sensitivity analysis.

| **Cohort** | **Normalization** | **n** | **AUC** | **Sensitivity** | **Specificity** | **Accuracy** | **Brier** |
| --- | --- | --- | --- | --- | --- | --- | --- |
| GSE64217 | cohort-wise z-score | 6 | 1.0000 | 1.0000 | 1.0000 | 1.0000 | 0.0304 |
| GSE6791 | cohort-wise z-score | 28 | 0.9938 | 0.9000 | 1.0000 | 0.9286 | 0.0561 |
| GSE7803 | cohort-wise z-score | 41 | 0.9167 | 0.7812 | 0.8889 | 0.8049 | 0.1306 |
| GSE64217 | frozen development scaling | 6 | 1.0000 | 1.0000 | 1.0000 | 1.0000 | 0.0134 |
| GSE6791 | frozen development scaling | 28 | 0.9875 | 0.9500 | 1.0000 | 0.9643 | 0.0531 |
| GSE7803 | frozen development scaling | 41 | 0.9132 | 0.7500 | 0.8889 | 0.7805 | 0.1299 |

# Supplementary Table S4. Feature-selection frequency across 50 outer models.

| **Gene** | **Selected outer models** | **Selection frequency** |
| --- | --- | --- |
| AKR1B1 | 50 | 100% |
| EED | 50 | 100% |
| FMO3 | 50 | 100% |
| SMPD2 | 50 | 100% |
| VEGFA | 50 | 100% |
| TNNI2 | 49 | 98% |
| NEU1 | 47 | 94% |
| GSTA1 | 44 | 88% |
| CD207 | 38 | 76% |
| TM7SF2 | 30 | 60% |
| NAAA | 23 | 46% |
| ADM | 22 | 44% |
| LBR | 22 | 44% |
| HEXA | 19 | 38% |
| SAT1 | 16 | 32% |
| BIRC3 | 15 | 30% |
| PLCD1 | 13 | 26% |
| FMO1 | 8 | 16% |
| OLR1 | 8 | 16% |
| STK3 | 7 | 14% |
| NT5C2 | 4 | 8% |
| SOAT1 | 2 | 4% |
| GALNT2 | 1 | 2% |
| GAMT | 1 | 2% |

# Supplementary File Note

Machine-readable prediction, fold-level, calibration, SHAP, and performance CSV files, together with the corrected R scripts and locked RDS model object, are supplied in the final submission directory.
