# Supplementary material for: A transparent 13-gene transcriptomic candidate signature for cervical neoplastic spectrum across GEO cohorts
Source: Front Genet. 2026 Oct 30;17:1875050. doi: 10.3389/fgene.2026.1875050 (PMC13759488; doi:10.3389/fgene.2026.1875050)
Supplement: Supplementary file 2 [file Table2.docx]

**Supplementary Tables S5-S7**

**Supplementary Table S5. Post hoc fixed-panel comparison in the 200-sample development matrix.**

| **Model** | **Genes** | **AUC (95% CI)** | **Sens.** | **Spec.** | **Brier** | **Intercept** | **Slope** | **ECE** | **ICI** |
| --- | --- | --- | --- | --- | --- | --- | --- | --- | --- |
| Fixed 13-gene panel | 13 | 0.924 (0.884-0.965) | 0.850 | 0.877 | 0.101 | -0.155 | 1.122 | 0.047 | 0.023 |
| Post hoc 5-gene core | 5 | 0.931 (0.895-0.966) | 0.961 | 0.753 | 0.096 | -0.085 | 1.044 | 0.063 | 0.010 |

Note: Results use 10 repeats of stratified five-fold outer validation, train-fold scaling, and inner GBM tree selection on the inherited globally ComBat-adjusted development matrix. The 5-gene core (SMPD2, VEGFA, AKR1B1, EED, FMO3) was defined from the prior 50-model selection-frequency audit. Paired DeLong comparison of the aggregated out-of-fold probabilities: P = 0.390. This is a post hoc sensitivity analysis, not an independently validated replacement model. ECE, 10-bin expected calibration error; ICI, loess-based integrated calibration index.

**Supplementary Table S6. Quantitative batch-separation quality-control indices.**

| **Stage** | **PC1 variance** | **PC2 variance** | **PC1+PC2 variance** | **Mean batch silhouette** |
| --- | --- | --- | --- | --- |
| Before ComBat | 0.533 | 0.125 | 0.658 | 0.552 |
| After ComBat | 0.218 | 0.123 | 0.341 | -0.011 |

Note: PCA was performed on the 500 most variable genes in the development matrix; silhouette values were calculated for GEO-series labels from the first 10 PCs. These descriptive values show reduced series separation after ComBat but do not prove preservation of biological signal, eliminate all confounding, or establish leakage-free end-to-end model validation.

**Supplementary Table S7. Differential-expression effect-size threshold sensitivity in the development cohort.**

| **\|log2FC\| threshold** | **Approx. fold change** | **FDR-significant genes** | **Up / down** | **13-panel genes meeting threshold** |
| --- | --- | --- | --- | --- |
| 0.585 | 1.5 | 1,532 | 811 / 721 | 13 / 13 |
| 1.000 | 2.0 | 559 | 280 / 279 | 4 / 13 |

Note: Counts were recalculated from the original limma output (10,783 tested genes) using FDR-adjusted P < 0.05. The 2-fold threshold retained AKR1B1, CD207, SMPD2, and TM7SF2 among the fixed 13-gene panel. This analysis evaluates the stringency of descriptive differential-expression screening only. The fixed 13-gene model was not reselected or retrained at either threshold, so these counts do not establish model robustness or a new classifier.
